# CLCA4 and MS4A12 as the significant gene biomarkers of primary colorectal cancer

**DOI:** 10.1042/BSR20200963

**Published:** 2020-08-20

**Authors:** Jing Han, Xue Zhang, Yan Liu, Li Jing, Yi-bing Liu, Li Feng

**Affiliations:** Department of Medical Oncology, The Fourth Hospital of Hebei Medical University, 12 Jiankang Road, Shijiazhuang, Hebei 050000, P.R. China

**Keywords:** bioinformatics, biomarker, hub genes, overall survival, primary colorectal cancer

## Abstract

Background: Primary colorectal cancer (PCRC) is a common digestive tract cancer in the elderly. However, the treatment effect of PCRC is still limited, and the long-term survival rate is low. Therefore, further exploring the pathogenesis of PCRC, and searching for specific molecular targets for diagnosis are the development trends of precise medical treatment, which have important clinical significance.

Methods: The public data were downloaded from Gene Expression Omnibus (GEO) database. Verification for repeatability of intra-group data was performed by Pearson’s correlation test and principal component analysis. Differentially expressed genes (DEGs) between normal and PCRC were identified, and the protein–protein interaction (PPI) network was constructed. Significant module and hub genes were found in the PPI network. A total of 192 PCRC patients were recruited between 2010 and 2019 from the Fourth Hospital of Hebei Medical University. RT-PCR was used to measure the relative expression of CLCA4 and MS4A12. Furthermore, the study explored the effect of expression of CLCA4 and MS4A12 for overall survival.

Results: A total of 53 DEGs were identified between PCRC and normal colorectal tissues. Ten hub genes concerned to PCRC were screened, namely CLCA4, GUCA2A, GCG, SST, MS4A12, PLP1, CHGA, PYY, VIP, and GUCA2B. The PCRC patients with low expression of CLCA4 and MS4A12 has a worse overall survival than high expression of CLCA4 and MS4A12 (*P*<0.05).

Conclusion: The research of DEGs in PCRC (53 DEGs, 10 hub genes, especially CLCA4 and MS4A12) and related signaling pathways is conducive to the differential analysis of the molecular mechanism of PCRC.

## Introduction

Primary colorectal cancer (PCRC) is a common digestive tract cancer in the elderly. The primary lesion can be seen in the left colon, the right colon, the upper or lower rectum [1,2]. PCRC is the second most commonly diagnosed cancer in women and the third most commonly diagnosed cancer in men, and the prevalence of male is higher than that of female in most areas [3,4]. With the social environment, lifestyle, and dietary structure changes, the incidence of PCRC is on the rise, and there is a trend of rejuvenation. This is a social issue worthy of attention [5]. At present, there is controversy about the pathogenesis of PCRC. It is generally believed that smoking, drinking, greasy diet, obesity, lack of exercise, colorectal inflammation, and genetic factors are all involved in the onset of cancer. But these factors are also the cause of many other tumors. Therefore, the specific etiological mechanism of PCRC has not yet been elucidated [6,7]. Some scholars believe that some genes or molecules are involved in the development of PCRC. These findings promote the research and treatment of PCRC [8–11]. At present, the treatment of PCRC includes traditional surgery, chemotherapy, radiotherapy, emerging immunotherapy, molecular targeted therapy, etc. Clinically, the single or combination therapy that best suits the condition is usually selected according to the actual situation of the patient [12,13]. However, the treatment effect of PCRC is still limited, and the long-term survival rate is low. The early prognosis of patients with early diagnosis is often better [14]. Therefore, further exploring the pathogenesis of PCRC, searching for specific molecular targets for diagnosis and treatment, realizing early diagnosis, targeted treatment and individualized treatment are the development trends of precise medical treatment, which have important clinical significance.

Personalized medicine refers to the treatment of existing diseases based on the information of each person’s disease genome [15]. It is now widely believed that majority of individual differences in drug response are due to genetic factors. Personalized medicine is a discipline that emphasizes studying the effect of genetic factors on a drug [16]. Recently, due to the smooth implementation of the human genome project and the rapid development of bioinformatics, personalized medicine has been strongly promoted, and the concept has been gradually developed [17].

Bioinformatics is a method to process and analyze biological data by combining biological knowledge with information processing technology. It is commonly used in high-throughput data analysis such as gene and proteomics. As a frontier interdisciplinary subject, bioinformatics analysis technology can realize the biological analysis of the structure and function of histological data, find the genes or proteins most relevant to diseases, and further analysis may find the molecules most relevant to diseases and can be used as disease markers [18,19]. At present, a large number of scholars have applied this technology to tumor research, that is, processing gene sequence or omics data by bioinformatics analysis technology to find genes or molecular markers most relevant to tumors [19–21].

Therefore, the present study aimed to use the bioinformatics to identify the hub genes of PCRC, and to verify their role on the overall survival of patients with PCRC based on the clinical data. And the research would provide novel insights for the personalized medicine on the treatment of patients with PCRC.

## Material and methods

### Lease start with dates and time, location of study, and the recruitments of patients

The present study recruited a total of 192 PCRC patients between 2010 and 2019 from the Fourth Hospital of Hebei Medical University, Shijiazhuang of Hebei province. Clinical and histopathological characteristics and follow-up and survival information were available for all patients, and were collected retrospectively from medical records. Patients aged 30–100 years old, histologically confirmed as PCRC, not received tumor treatment, and no history of gastrointestinal surgery will be screened for inclusion criteria. Exclusion criteria included: age <30 years old or >100 years old, combined with other malignant tumors, operation time more than one month after the last examination, and severs heart disease.

### Ethical clearance and informed content

The research conformed to the Declaration of Helsinki and was authorized by the Human Ethics and Research Ethics Committees of the Fourth Hospital of Hebei Medical University. The written informed consents were obtained from all participates.

### Download public data

The Gene Expression Omnibus (GEO) database [22] (http://www.ncbi.nlm.nih.gov/geo) is the largest, most comprehensive, and publicly available source of gene expression data. It contains information about the expression levels of multiple genes in different groups of clinical samples, such as the differences in gene expression between tumor tissues and normal tissues. GSE41258 (GPL96 [HG-U133A] Affymetrix Human Genome U133A Array) and GSE81558 (GPL15207 [PrimeView] Affymetrix Human Gene Expression Array) were obtained from the GEO database. A total of 240 samples, including 186 tumor colorectum tissues from PCRC patients and 54 normal colorectum tissues, were selected from GSE41258. A total of 32 samples, including 23 tumor colorectum tissues from PCRC patients and 9 normal colorectum tissues, were selected from GSE81558.

### Verification for repeatability of intra-group data

First, repeatability of intra-group data were verified by the Pearson’s correlation test. The heatmap was drew via the R language environment, and presented the correlation among intra-group data. Second, principal component analysis (PCA) was the general method for sample clustering, and is commonly performed for diversity analysis, resequencing, gene expression, and other sample clustering based on various variable information. The verification for repeatability of intra-group data was executed by PCA.

### Differentially expressed genes (DEGs) between normal and PCRC

GEO2R [23] (http://www.ncbi.nlm.nih.gov/geo/geo2r) could import data of the GEO database into the R language and perform differential analysis, essentially through the following two R packages, including limma packages and GEOquery. Therefore, through the GEO2R tool, DEGs were identified between normal and PCRC group. The adjusted *P*-values (adj. *P*) <0.05, and the fold change (FC) ≥ 1.5 or ≤ −1.5 were defined as significance. SangerBox (https://shengxin.ren), one open tool, was used to draw volcano maps [24]. Venn diagrams were delineated using an online Venn tool (http://bioinformatics.psb.ugent.be/webtools/Venn/), which would visualize common DEGs shared between GSE41258 and GSE81558.

### Protein–protein interaction (PPI) network

The common DEGs, shared between GSE41258 and GSE81558, were converted into differently expressed proteins. The STRING (Search Tool for the Retrieval of Interacting Genes) online database (http://string-db.org) could construct PPI network, which was visualized by Cytoscape (version 2.8) [25].

### GO and KEGG analysis via DAVID tool

One online tool, DAVID (https://david.ncifcrf.gov/home.jsp) (version 6.8, Maryland, America), was applied to carried out the functional annotation for DEGs. Gene Ontology (GO) [26] generally perform enrichment analysis of genomes. And there are mainly cellular components (CC), biological processes (BP), and molecular functions (MF) in the GO analysis. Kyoto Encyclopedia of Genes and Genomes (KEGG) (https://www.kegg.jp/) [27] is a comprehensive database of genomic, chemical, and systemic functional information. Therefore, DAVID was used to make analysis of GO and KEGG.

### Significant module and hub genes

Molecular Complex Detection tool (MCODE) (version 1.5.1) [28], an open plug-in of Cytoscape, was performed to identify tested most significant module from the PPI network, and the criteria was that the maximum depth = 100, MCODE scores >5, cut-off = 2, *k*-score = 2, and node score cut-off = 0.2.

Then, cytoHubba [29], a free plug-in of Cytoscape, was applied to authorize the hub genes, when the degree ≥10. Chia-Hao Chin’s [29] research introduce a novel Cytoscape plugin cytoHubba for ranking nodes in a network by their network features. CytoHubba provide a user-friendly interface to explore important nodes in biological networks. When the degree ≥10 in the cytoHubba, the 10 hub genes would be obtained. And in the former publications [30–32], numerous researchers chose 10 hub genes out of the DEGs. Therefore, the present study chose 10 hub genes out of 53 DEGs.

### Interaction between the hub genes

Pearson’s correlation analysis was also performed to present the interaction between the hub genes. The cBioPortal (http://www.cbioportal.org) [33], one online software, constructed the co-expression network of these hub genes. Simultaneously, the co-expression network of hub genes in the field of PCRC was also analyzed via Coexpedia, a free and open online tool(http://www.coexpedia.org/) [34].

### Expression analysis of hub genes

UCSC Xena (https://xena.ucsc.edu/welcome-to-ucsc-xena/) could integrate the public genomic data sets to analyze and visualize the expression level of hub genes. Then, the clustering analysis of expression level of hub genes was performed using heatmaps based on the GSE41258 and GSE81558. Also, the expression profiles of hub genes in the human different organs were displayed with Gene Expression Profiling Interactive Analysis (GEPIA, http://gepia.cancer-pku.cn/) [35]. In order to compare the expression of hub genes in the various tumors, GEPIA was used. And the expression profiles of hub genes in the PCRC and normal groups were analyzed using GEPIA.

### Effect of hub gene expression for pathological stage and overall survival

Effect of hub gene expression for pathological stage and overall survival was analyzed by the GEPIA. Finally, the correlation and linear regression analysis between PCRC and hub gene expression were performed. And the receiver operator characteristic (ROC) curve analysis was performed to test the sensitivity and specificity of hub gene expression for diagnose PCRC. The SPSS software (version 21.0; IBM; New York; America) was used to conduct all the statistical analysis. A *P*-value <0.05 was defined as statistical significance.

### RT-qPCR assay

Total RNA was extracted from tumor colorectum tissues from PCRC patients and adjacent normal colorectum tissues by the RNAiso Plus (Trizol) kit (Thermofisher, Massachusetts, America), and reverse transcribed to cDNA. RT-qPCR was performed using a Light Cycler® 4800 System with specific primers for the ten hub genes. Table 1 presents the primer sequences used in the experiments. The RQ values (2−ΔΔ*C*t, where *C*t is the threshold cycle) of each sample were calculated, and are presented as fold change in gene expression relative to the control group. GAPDH was used as an endogenous control. The expression level of CLCA4 and MS4A12 in PCRC patients was measured by RT-qPCR.

**Table 1:** Summaries for the function of 10 hub genes

No.	Gene symbol	Full name	Function
1	CLCA4	Chloride channel accessory 4	May be involved in mediating calcium-activated chloride conductance
2	GUCA2A	Guanylate cyclase activator 2A	Endogenous activator of intestinal guanylate cyclase. It stimulates this enzyme through the same receptor binding region as the heat-stable enterotoxins
3	GCG	Glucagon	Regulates blood glucose by increasing gluconeogenesis and decreasing glycolysis. GLP-1 is a potent stimulator of glucose-dependent insulin release. GLP-2 stimulates intestinal growth, concomitant with increased crypt cell proliferation
4	SST	Somatostatin	Somatostatin inhibits the release of somatotropin. This hormone is an important regulator of the endocrine system through its interactions with pituitary growth hormone, thyroid stimulating hormone, and most hormones of the gastrointestinal tract
5	MS4A12	Membrane spanning 4-domains A12	May be involved in signal transduction as a component of a multimeric receptor complex. Silencing of this gene in colon cancer cells inhibits the proliferation, cell motility, and chemotactic invasion of cells
6	PLP1	Proteolipid protein 1	This is the major myelin protein from the central nervous system. It plays an important role in the formation or maintenance of the multilamellar structure of myelin
7	CHGA	Chromogranin A	This gene product is a precursor to three biologically active peptides; vasostatin, pancreastatin, and parastatin
8	PYY	Peptide YY	This gut peptide inhibits exocrine pancreatic secretion, has a vasoconstrictory action, and inhibitis jejunal and colonic mobility
9	VIP	Vasoactive intestinal peptide	VIP causes vasodilation, lowers arterial blood pressure, stimulates myocardial contractility, increases glycogenolysis and relaxes the smooth muscle of trachea, stomach and gallbladder
10	GUCA2B	Guanylate cyclase activator 2B	May be a potent physiological regulator of intestinal fluid and electrolyte transport. May be an autocrine/paracrine regulator of intestinal salt and water transport

### Overall survival analysis of the PCRC

The Kaplan–Meier method was performed to analyze the overall survival. All statistical analyses were conducted using SPSS software (version 21.0), and *P*<0.05 was considered statistically significant.

## Results

### High repeatability of data

There exist strong correlations among samples in the PCRC group, and also strong correlations among samples in the control group in the GSE41258 via the Pearson’s correlation test (Supplementary Figure S1A). And there also exist strong correlations among samples in the PCRC group, and also strong correlations among samples in the control group in the GSE81558 via the Pearson’s correlation test (Supplementary Figure S1B). Furthermore, PCA was performed to verify the repeatability of data. Through the PCA, the repeatability of the data in GSE41258 was fine. The distances between per samples in the PCRC group were close, and the distances between per samples in the control group were also close in the dimension of PC1 (Supplementary Figure S1C). Through the PCA, the repeatability of the data in GSE81558 was fine. The distances between per samples in the PCRC group were close, and the distances between per samples in the control group were also close in the dimension of PC1 (Supplementary Figure S1D).

### DEGs between control and PCRC

**Figure 1:**
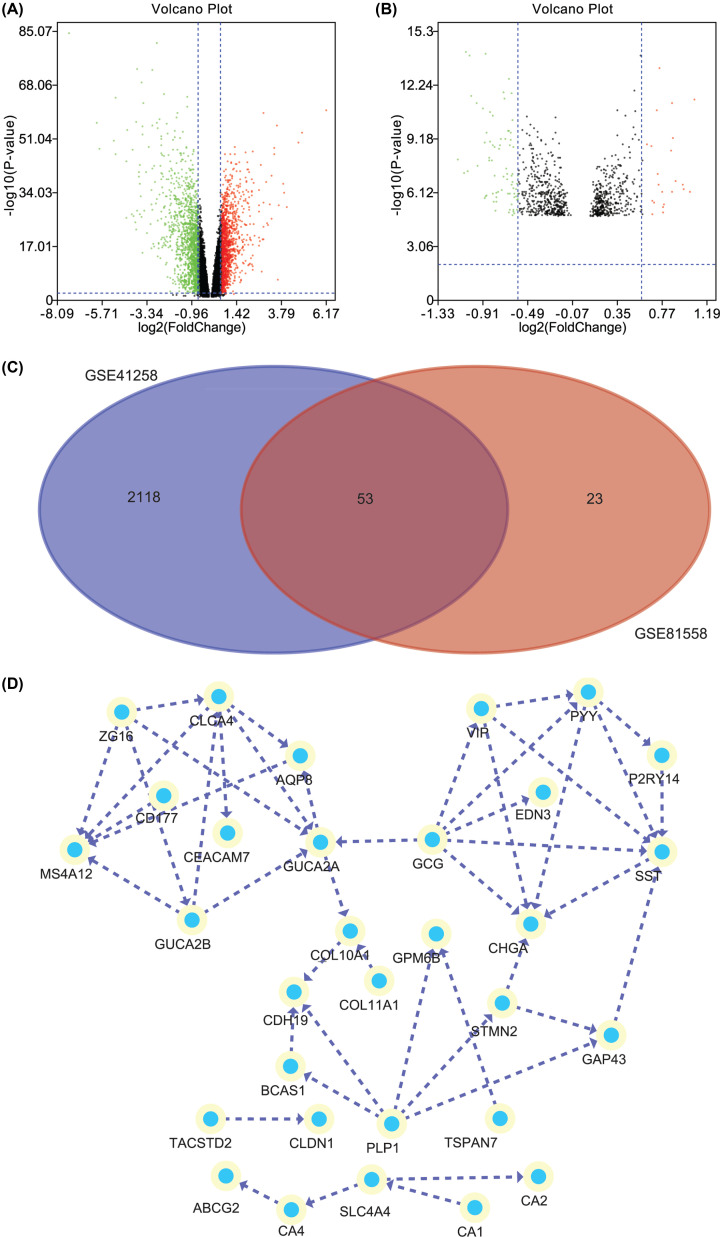
The differently expressed genes and PPI network (**A**) One volcano plot presents the DEGs in the GSE41258. (**B**) Another volcano plot presents the DEGs in the GSE81558. In the volcano plots, the green nodes indicate the down-regulated DEGs, and the red nodes indicate the up-regulated DEGs. (**C**) The Venn diagram manifested that a total of 53 DEGs were exist in the two datasets (GSE41258 and GSE81558) simultaneously. (**D**) The PPI network of the common DEGs.

There are plenty of DEGs on the all chromosomes between PCRC and control samples (Supplementary Figure S1E). One volcano plot presents the DEGs in the GSE41258 (Figure 1A), and another volcano plot presents the DEGs in the GSE81558 (Figure 1B). In the volcano plots, the green nodes indicate the down-regulated DEGs, and the red nodes indicate the up-regulated DEGs. The Venn diagram manifested that a total of 53 DEGs were exist in the two datasets (GSE41258 and GSE81558) simultaneously (Figure 1C). After construction of PPI network for the common DEGs, there are 31 nodes and 46 edges in the PPI network (Figure 1D).

### The functional enrichment analysis of DEGs via GO and KEGG

**Figure 2:**
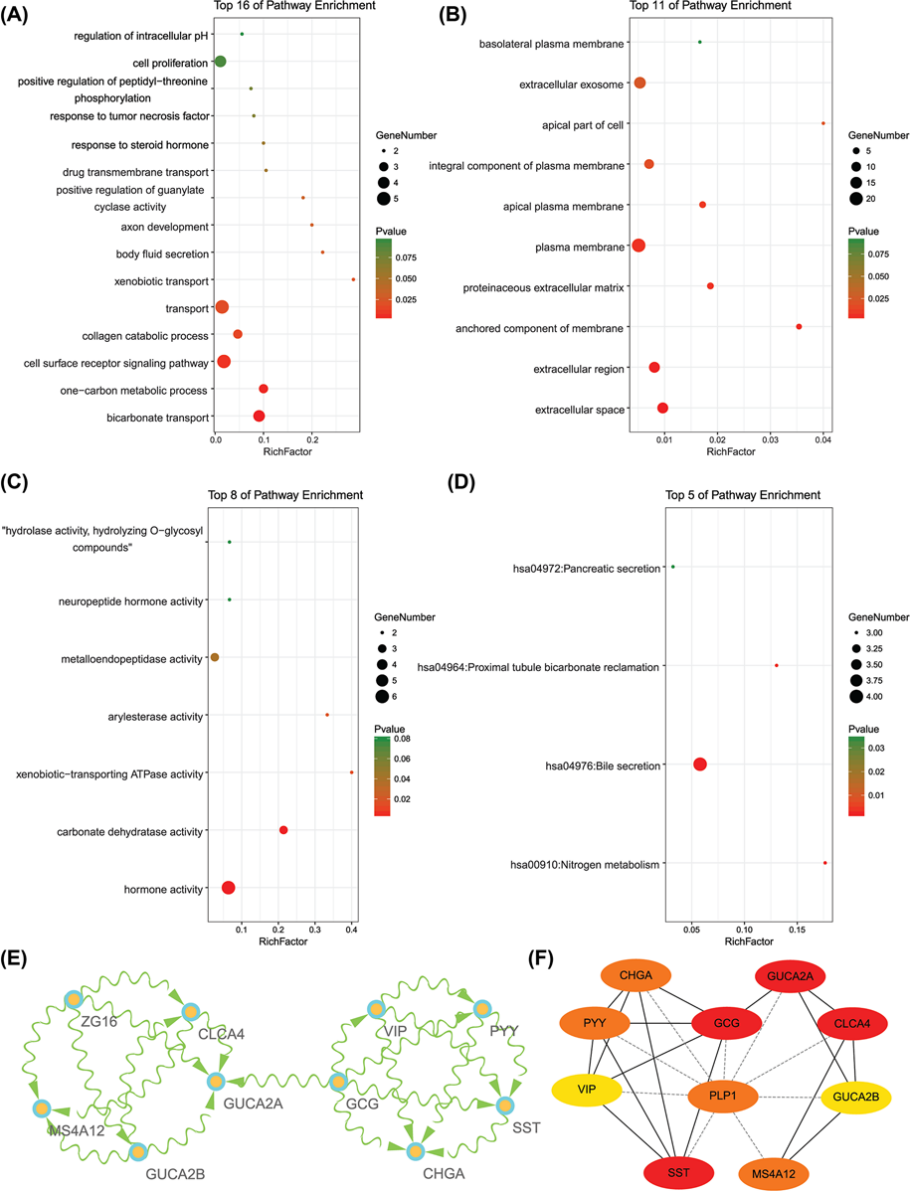
The enrichment analysis for DEGs and the identification of hub genes (**A**) Detailed information relating to changes in the biological processes (BP) of DEGs in PCRC and control colorectal samples. (**B**) Detailed information relating to changes in the cellular components (CC) of DEGs in PCRC and control colorectal samples. (**C**) Detailed information relating to changes in the molecular functions (MF) of DEGs in PCRC and control colorectal samples. (**D**) KEGG pathway analysis for DEGs. (**E**) The significant module of the PPI network. (**F**) The hub genes identified from the PPI network.

GO analysis manifested that variations in DEGs related with biological processes (BP) were significantly enriched in bicarbonate transport, one-carbon metabolic process, cell surface receptor signaling pathway, collagen catabolic process, transport, xenobiotic transport, body fluid secretion, axon development, positive regulation of guanylate cyclase activity, drug transmembrane transport, response to steroid hormone, response to tumor necrosis factor, positive regulation of peptidyl–threonine phosphorylation, cell proliferation, and regulation of intracellular pH (Figure 2A). The variations in DEGs related with cellular components (CC) were significantly enriched in extracellular space, extracellular region, anchored component of membrane, proteinaceous extracellular matrix, plasma membrane, apical plasma membrane, integral component of plasma membrane, apical part of cell, extracellular exosome, and basolateral plasma membrane (Figure 2B). The variations in DEGs related with molecular functions (MF) were significantly enriched in hormone activity, carbonate dehydratase activity, xenobiotic-transporting ATPase activity, arylesterase activity, metalloendopeptidase activity, neuropeptide hormone activity, and ‘hydrolase activity, hydrolyzing O-glycosyl compounds’ (Figure 2C). KEGG pathway enrichment analysis showed that the top pathways related with DEGs were nitrogen metabolism, bile secretion, proximal tubule bicarbonate reclamation, and pancreatic secretion (Figure 2D).

### Significant module network and identification of hub genes

A significant module was screened from the PPI network, and the module network consisted of 10 nodes and 20 edges (Figure 2E). And ten hub genes were identified, including CLCA4, GUCA2A, GCG, SST, MS4A12, PLP1, CHGA, PYY, VIP, and GUCA2B (Figure 2F). The function of 10 hub genes were summarized in the Table 1.

### Strong interaction among the hub genes

Through the Pearson’s correlation test, heat maps manifested that there were strong correlations among hub genes in the GSE41258 (Supplementary Figure S2A) and GSE81558 (Supplementary Figure S2B) datasets. PYY, SST, GCG, and VIP existed simultaneously in the co-expression network via cBioPortal (Supplementary Figure S2C). And through the analysis of Coexpedia, there were strong interactions among PYY, SST, GCG, CHGA, CLCA4, GUCA2B, and MS4A12 (Supplementary Figure S2D).

### Difference of expression of hub genes between PCRC and control samples

Heat map showed that the expressions of all the hub genes were lower in the PCRC samples than the control samples (Supplementary Figure S2E). Hierarchical clustering allowed for simple differentiation of PCRC tissues from normal colorectal tissues via the expression levels of hub genes in the GSE41258 (Supplementary Figure S3A) and GSE81558 (Supplementary Figure S3B) datasets. The expressions of all the hub genes were lower in the PCRC group than the control group.

### The analysis of expression level of hub genes

The hub genes in the human different organs were expressed in the Supplementary Figure S3C. The pink presents the tumor individuals, and the green presents the normal individuals. The expression of hub genes in the colorectum was higher in the normal individuals compared with the tumor samples (Supplementary Figure S3C). When we compared the expression of hub genes in the various tumors, the all hub genes were down-regulated in the PCRC samples, also named colon adenocarcinoma (COAD) (Supplementary Figure S3D). Through GEPIA analysis, the expressions of hub genes in the PCRC patients were lower than the normal individuals (Supplementary Figure S4A).

### Association between hub gene expression, pathological stage, and overall survival

**Figure 3:**
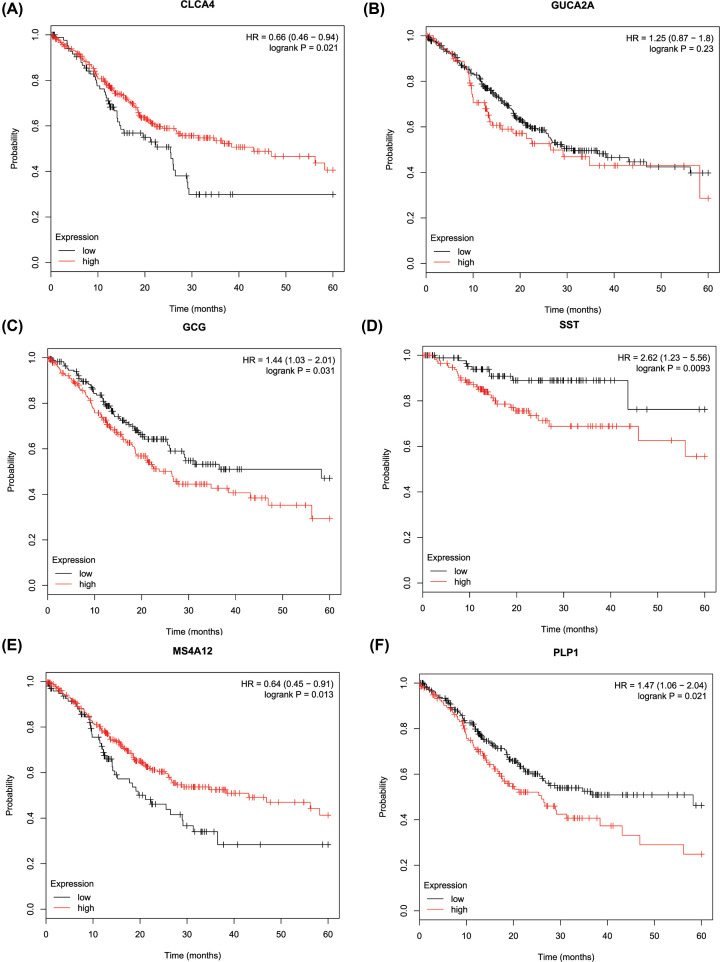
The overall survival Kaplan–Meier of six hub genes (**A**) CLCA4, (**B**) GUCA2A, (**C**) GCG, (**D**) SST, (**E**) MS4A12, (**F**) PLP1.

The results of GEPIA manifested that the expression of VIP was significantly positively related with pathological stage (*P*=0.027), while the expression of CLCA4, GUCA2A, GCG, SST, MS4A12, PLP1, CHGA, PYY, and GUCA2B was not as (Supplementary Figure S4B). Kaplan–Meier analysis showed that PCRC patients with low expression levels of CLCA4, and MS4A12 had poorer overall survival times than those with high expression levels (*P*<0.05; Figure 3A,E). PCRC patients with high expression levels of GCG, SST, PLP1, and CHGA had poorer overall survival times than those with low expression levels (*P*<0.05; Figure 3C,D,F; Supplementary Figure S5A). However, there was no statistically significant effect on OS associated with the expression of GUCA2A, PYY, VIP, and GUCA2B (*P*>0.05; Figure 3B, Supplementary Figure S5B–D).

### Correlation, linear regression, and ROC analysis

The Pearson’s correlation coefficient was used in the correlation analysis, and CLCA4 (*ρ* = −0.868, *P*<0.001), GUCA2A (*ρ* = −0.837, *P*<0.001), GCG (*ρ* = −0.726, *P*<0.001), SST (*ρ* = −0.616, *P*=0.001), MS4A12 (*ρ* = −0.793, *P*<0.001), PLP1 (*ρ* = −0.763, *P*<0.001), CHGA (*ρ* = −0.634, *P*<0.001), PYY (*ρ* = −0.610, *P*<0.001), VIP (*ρ* = −0.600, *P*<0.001), and GUCA2B (*ρ* = −0.688, *P*<0.001) were significantly correlated with PCRC (Table 2). In the multivariate linear regression model, holding all other variables at a fixed value, the natural logarithmic DN remained associated with CLCA4, GUCA2A, SST, MS4A12, PLP1, CHGA, PYY, and GUCA2B (*P*<0.05) (Table 2).

**Table 2:** The correlation and linear regression analysis between PCRC and relevant gene expression

	PCRC			
Gene symbol	Pearson’s correlation coefficient		Multiple linear regression	
	*ρ*^a^	*P*-value	*P*-value	VIF
CLCA4	−0.868	<0.001***	<0.001***	6.959
GUCA2A	−0.837	<0.001***	<0.001***	8.947
GCG	−0.726	<0.001***	0.087	4.944
SST	−0.616	0.001**	0.017*	3.755
MS4A12	−0.793	<0.001***	0.039*	4.685
PLP1	−0.763	<0.001***	<0.001***	2.363
CHGA	−0.634	<0.001***	0.001**	2.290
PYY	−0.610	<0.001***	0.004**	2.195
VIP	−0.600	<0.001***	0.271	2.157
GUCA2B	−0.688	<0.001***	0.017*	4.144

a Pearson’s correlation coefficient between PCRC and relevant characteristics; *ρ*: Pearson’s correlation coefficient.

b Multiple linear regression analysis; PCRC: primary colorectal cancer.

* : *P*<0.05;

** : *P*<0.01;

*** : *P*<0.001

**Figure 4:**
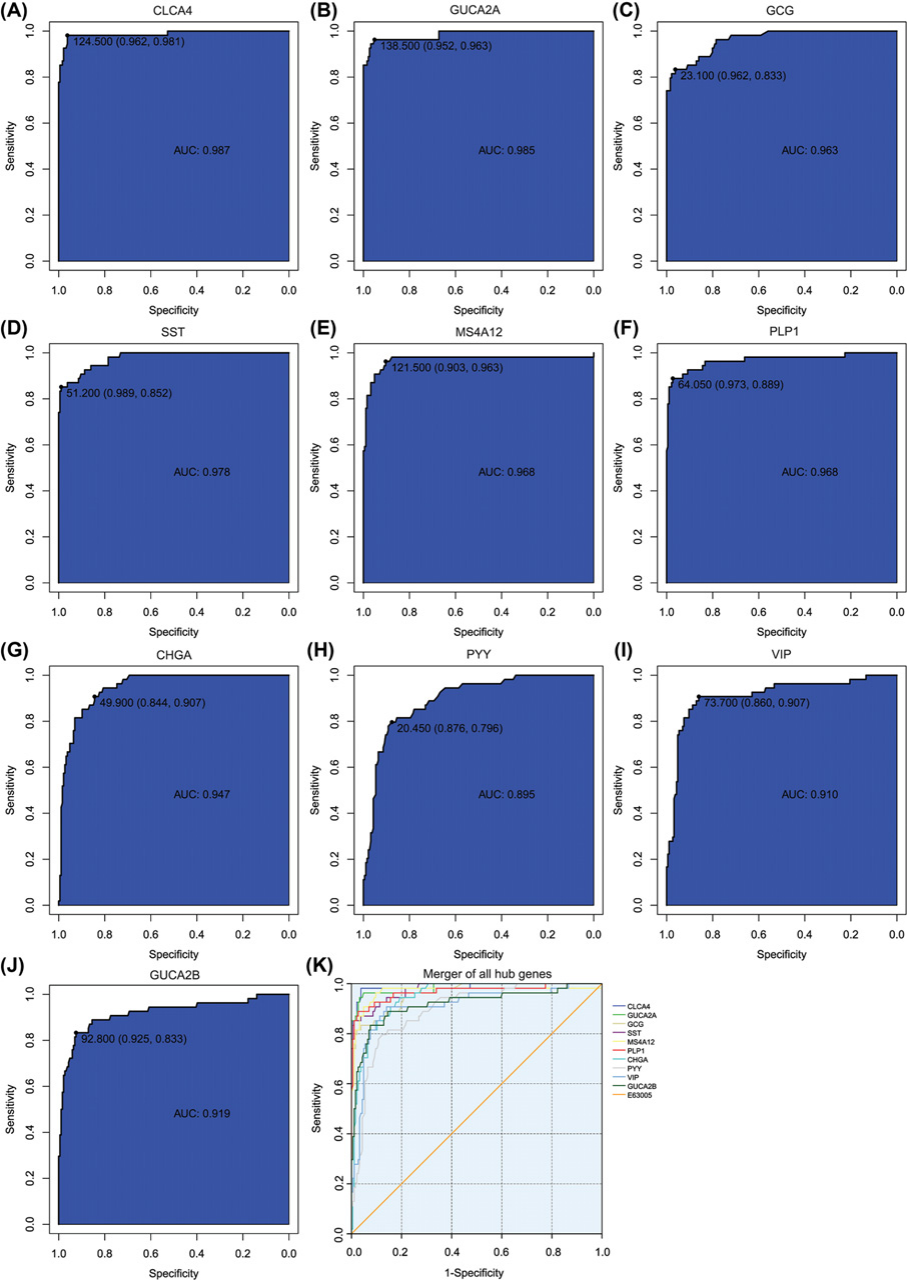
ROC curves of hub genes for PCRC (**A**) CLCA4, (**B**) GUCA2A, (**C**) GCG, (**D**) SST, (**E**) MS4A12, (**F**) PLP1, (**G**) CHGA, (**H**) PYY, (**I**) VIP, (**J**) GUCA2B, (**K**) ROC curves of all hub genes.

To identify accurate thresholds for hub genes to predict PCRC, we constructed ROC. The expression of all hub genes was associated with a diagnosis of PCRC (0.890 < AUC < 1, *P*-value<0.001) (Table 3 and Figure 4). The ROC curve of CLCA4 was shown in Figure 4A, and the area under curve of CLCA4 was maximal. The ROC curve of GUCA2A was shown in Figure 4B. The ROC curve of GCG was shown in Figure 4C. The ROC curve of SST was shown in Figure 4D. The ROC curve of MS4A12 was shown in Figure 4E. The ROC curve of PLP1 was shown in Figure 4F. The ROC curve of CHGA was shown in Figure 4G. The ROC curve of PYY was shown in Figure 4H. The ROC curve of VIP was shown in Figure 4I. The ROC curve of GUCA2B was shown in Figure 4J. The ROC curves of per hub genes are shown in Figure 4K.

**Table 3:** Receiver operator characteristic curve analysis of hub gene expression for PCRC

Gene symbol	PCRC			
	AUC	*P*-value	95%CI	ODT
CLCA4	0.987^max^	<0.001***	0.962–0.981	124.500
GUCA2A	0.985	<0.001***	0.952–0.963	138.500
GCG	0.963	<0.001***	0.833–0.962	23.100
SST	0.978	<0.001***	0.852–0.989	51.200
MS4A12	0.968	<0.001***	0.903–0.963	121.500
PLP1	0.968	<0.001***	0.889–0.973	64.050
CHGA	0.947	<0.001***	0.844–0.907	49.900
PYY	0.895	<0.001***	0.796–0.876	20.450
VIP	0.910	<0.001***	0.860–0.907	73.700
GUCA2B	0.919	<0.001***	0.833–0.925	92.800

AUC: area under curve; ^max^ the maximum of AUC; *Significant variables; ODT: Optimal diagnostic threshold;

PCRC: primary colorectal cancer.***: *P*<0.001.

### Basic information of PCRC patients

Patients’ basic information were presented in Table 4. The mean patient age was 66 years old (range, 35–96 years), and the median OS was 52 months (range, 5–108 months).

**Table 4:** Clinicopathological variables and the expression status of CLCA4 and MS4A12

		CLCA4		*P*	MS4A12		*P*
		Low (%)	High (%)		Low (%)	High (%)	
Sex							
Male	161	77(40.1%)	84(43.8%)	0.007*	73(38.0%)	88(45.8%)	0.001*
Female	31	23(12.0%)	8(4.2%)		24(12.5%)	7(3.6%)	
Age							
<65 years	88	44(22.9%)	44(22.9%)	0.595	38(19.8%)	50(26.0%)	0.061
≥65 years	104	56(29.2%)	48(25.0%)		59(30.7%)	45(23.4%)	
Overall survival							
<60 months	122	90(46.9%)	32(16.7%)	<0.001*	81(42.2%)	41(21.4%)	<0.001*
≥60 months	70	10(5.2%)	60(31.3%)		16(8.3%)	54(28.1%)	

Pearson’s chi-squared test was used.

* *P*<0.05

### RT-qPCR analysis validation of hub genes

**Figure 5:**
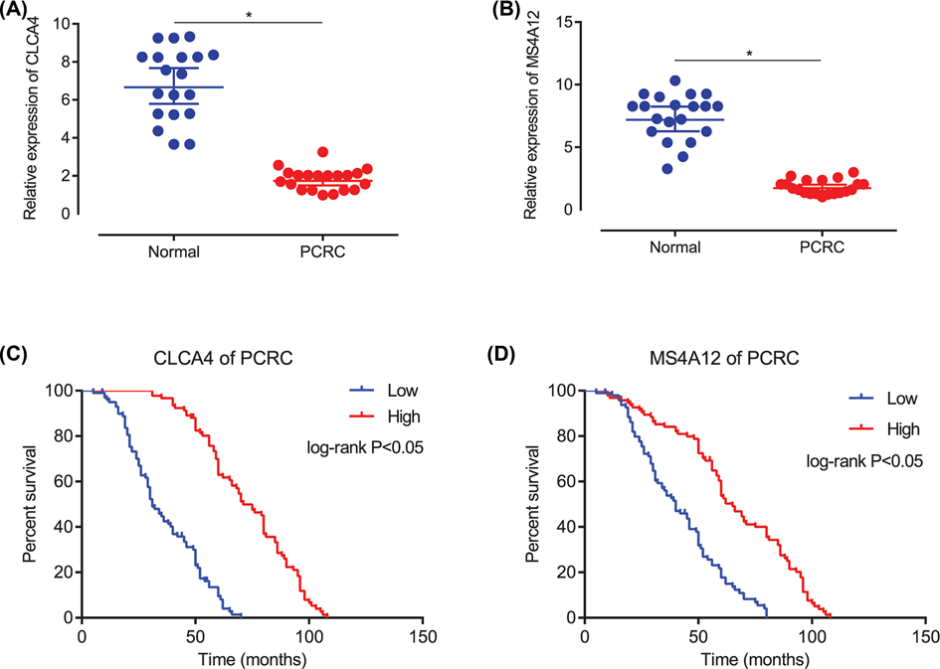
The verification of expression and overall survival analysis for CLCA4 and MS4A12 (**A**) The relative expression of CLCA4 based on PCR. (**B**) The relative expression of MS4A12 based on PCR. (**C**) The overall survival of PCRC based the expression of CLCA4. (**D**) The overall survival of PCRC based the expression of MS4A12.

As presented in the result, CLCA4 (*P*<0.05, Figure 5A) and MS4A12 (*P*<0.05, Figure 5B) were markedly down-regulated in PCRC samples, when compared with the adjacent normal colorectum tissues. It should be noted that the expression situation of CLCA4 and MS4A12 were consistent in above results of bioinformatics.

### Low expression of CLCA4 and MS4A12 in PCRC patients were independent prognostic factors for the poor overall survival

The Kaplan–Meier OS curves were analyzed. Low expression of CLCA4 was predictive of a shorter OS in the PCRC patients (*P*<0.05, Figure 5C). Low expression of MS4A12 was predictive of a shorter OS in the PCRC patients (*P*<0.05, Figure 5D).

## Discussion

PCRC is a common digestive tract cancer, which seriously affects the life expectancy and quality of life of patients. In recent years, the survey results show that the morbidity and mortality rate are on the rise [36]. The clinical manifestations of patients with PCRC are related to the location and pathological type of the tumor. The most common type of pathology is adenocarcinoma. The primary lesion located in the colon often causes diarrhea, obstruction, bleeding in the rectum, anemia, and cachexia in the later stage of cancer patients [37]. The current treatment is mainly surgery combined with chemotherapy or radiotherapy, while advocated exercise to enhance the body’s immunity and prevent infection [12]. Gavrilas et al. found that combination of dietary preparations such as curcumin and resveratrol with chemotherapeutic drugs contributed to the prognosis of PCRC [38]. Clinical application benefit and safety of epidermal growth factor EGFR-related targeted therapy and PD-1/PD-L1 immunotherapy are still to be further studied [39,40]. The investigation found that the cost of PCRC treatment is high, and it takes up a lot of medical resources, and the prognosis of patients is not necessarily proportional to the input. The early treatment of early treatment patients has a relatively low total cost of treatment and a good prognosis [41,42]. Therefore, to further explore the pathogenesis of PCRC, to find possible therapeutic targets, to achieve early diagnosis, targeted therapy, individualized treatment has important clinical value and market prospects.

Bioinformatics has been widely used as a new means of exploring disease mechanism and searching for disease-related genetic molecules. Zhang et al. found genes related to hepatocellular carcinoma by bioinformatics analysis. Further analysis confirmed the correlation between these differential genes and diseases, suggesting that these molecules may be used as molecular targets for early diagnosis and treatment [20]. Zhang et al. found the most relevant molecules of gastric cancer, miR-19b-3p and miR-16-5p, by analyzing the genome-wide miRNA microarray data of gastric cancer patients, which provided a new idea for the diagnosis and treatment of gastric cancer [43]. Sun found molecules related to the pathogenesis of colorectal cancer by screening from public databases. Further analysis showed that differentially expressed genes such as PPBP, CCL28, and CXCL12 are likely to be involved in the development of colorectal cancer and may be potential diagnostic and therapeutic targets [44]. We found 10 genes that were differentially expressed in patients with PCRC by bioinformatics analysis. Low expression of PLP1, VIP, SST, GCG, PYY, MS4A12, CLCA4, GUCA2A, CHGA, and GUCA2B in tumor patients compared with normal subjects. At the same time, we performed survival analysis on patients with PCRC. The results showed that CLCA4, GUCA2A, GCG, SST, MS4A12, and PLP1 genes were significantly associated with the survival of patients with PCRC.

CLCA4 is the chloride channel accessory 4. CLCA4 is a member of the calcium-sensitive chloride-transporting protein family involved in intracellular ion channel activity, chloride ion transmembrane transport, and proteolysis. Members of the calcium activated chloride channel (CLCA) gene family are thought to have multiple functions, including cell adhesion and tumor suppression. Ye et al. found that CLCA4 is low expressed in patients with oral tongue squamous cell carcinoma through genome-wide transcriptional mapping, which provides ideas for diagnosis and targeted therapy [45]. Bundela also found multiple differentially expressed genes in oral cancer patients in India, and suggested that CLCA4 may be a potential therapeutic target [46]. Yu et al. found that CLCA4 is low expressed in breast cancer patients. Further analysis revealed that CLCA4 is a marker of breast epithelial differentiation and may be involved in tumor proliferation and metastasis. Clinical data analysis showed that patients with breast cancer with low expression of CLCA4 had lower recurrence-free survival rate, suggesting that it may serve as a diagnostic and therapeutic target [47]. Hu found that CLCA4 was low expressed in bladder cancer tissues. Further analysis revealed that CLCA4 may be involved in the proliferation and invasion of bladder cancer through PI3K/AKT signal transduction, suggesting that CLCA4 may be a target for diagnosis and treatment [48]. Liu found that CLCA4 may inhibit epithelial–mesenchymal transition (EMT) by affecting PI3K/ATK phosphorylation, thereby inhibiting cell migration and invasion of hepatoma cells [49]. Yang found that patients with colorectal cancer CRC had low expression of CLCA1 and CLCA4, and further experiments confirmed that CLCA1 is involved in tumor proliferation and invasion [50]. Zhao found that CLCA4 was low expressed in colorectal patients [51]. Chen also found that CLCA4 was low expressed in patients with colorectal cancer, and believed that CLCA4 inhibited the epithelial–mesenchymal transformation of colorectal cancer through PI3K/ATK signaling pathway, thus participating in the proliferation and invasion of tumors, and may be used as a marker for diagnosis and judgment [52]. Consistent with the above results, we found that CLCA4 was lowly expressed in primary colorectal patients by bioinformatics analysis, and survival analysis of primary colorectal patients found that patients with low expression of CLCA4 had a worse prognosis in survival, suggesting the protective effect of CLCA4 on PCRC patients and its inhibitory effect on cancer. We speculated that CLCA4 affects epithelial–mesenchymal transition and intercellular communication via PI3K/ATK and participates in the development of primary colorectal patients, which may be potential diagnostic and therapeutic targets.

MS4A12 is membrane spanning 4-domains A12. As a cell protein, MS4A12 participates in cell membrane composition, cell differentiation, proliferation, and cell cycle regulation. Members of the MS4A family are likely to be part of the oligomeric cell surface complex, which has different signal transduction functions [53]. MS4A12 may promote the proliferation and invasion of colorectal cancer cells by influencing epidermal growth factor receptor. Further studies found that intestinal specific transcription factor CDX2 mediated by RNA interference (RNAi) is a trans-activator of growth-promoting gene expression in colorectal cancer, suggesting that MS4A12 may be a potential therapeutic target for colorectal cancer [54]. There was heterogeneity in cancer cells, and found that MS4A12 and other genes could be used to predict cancer patients, suggesting that MS4A12 might be a potential diagnostic target [55]. Multiple genes can be used as molecular markers to distinguish between colon adenomatous polyps and cancer, and that MS4A12 can be used as an early diagnostic target for colorectal cancer [56]. He found that MS4A12 participated in the differentiation of colon cancer cells. Survival analysis showed that patients with low expression of MS4A12 had a poor survival, suggesting that MS4A12 might be a molecular marker for diagnosis and prognosis [57]. We found that MS4A12 was low expressed in PCRC patients by bioinformatics analysis. Meanwhile, survival analysis of PCRC patients showed that patients with low expression of MS4A12 had worse survival than those with high expression of MS4A12, suggesting that MS4A12 was involved in the occurrence and development of PCRC and could inhibit the progression of cancer. We speculate that MS4A12 may be involved in the development of PCRC by affecting cell proliferation, differentiation and signaling pathway transduction, and may be a potential target for diagnosis and treatment.

Personalized medicine is based on the individual’s genetic structure to select the appropriate drug type and dose for patients to significantly improve the efficacy of drugs and reduce drug toxicity [15]. The research found that the expression of CLCA4 and MS4A12 were lower in the patients with PCRC than the normal individual, and overall survival analysis manifested that the PCRC patients with lower expression of CLCA4 and MS4A12 had poorer overall survivals. Therefore, CLCA4 and MS4A12 might be cancer suppressors which could be beneficial to the progression of patients with PCRC. Based on the principle of personalized medicine, the researchers could develop and popularize the drugs targeted to the CLCA4 and MS4A12 to suppress the occurrence and development of the PCRC. Detection of CLCA4 and MS4A12 expression might also help to identify risk factors with poor prognosis in patients with PCRC [58]. Therefore, assessing the level of CLCA4 and MS4A12 could help to accurately predict the prognosis, recurrence and the potential for secondary surgery, so that each patient can be treated individually and the therapeutic effect could be optimized [59]. Currently, the European and American countries have translated the experimental results of pharmacogenomics into clinical applications [60].

## Limitations

Despite the rigorous analysis process, there are still some deficiencies in this study. First, there are no animal experiments to comprehensively verify the accuracy of the results. Second, the genetic information that can be used to guide personalized medicine still needs to be enriched, and the basic research needs to be strengthened. Finally, a successful genetic test for personalized medicine meets two criteria for clinical application: safety and effectiveness. However, the bioinformatics in this research focuses on gene screening, and does not verify its safety and effectiveness.

## Conclusion

CLCA4 and MS4A12 might play a significant role in the development and survival of PCRC, and might eventually become biomarkers to the treatment of patients with PCRC. Detection of CLCA4 and MS4A12 in the clinical practice might provide the better evidence to guide the early diagnosis and treatment of PCRC. In the future, diagnostic reagent kit of CLCA4 and MS4A12 and the corresponding drugs should be developed to perform the multicenter randomized controlled clinical trial, which will provide new evidence and insights for exploring the early diagnosis of PCRC and personalized medicine.

## Supplementary Material

Supplementary Figures S1-S5

## Data Availability

The datasets used and/or analyzed during the current study are available from the corresponding author on reasonable request.

## Abbreviations

BP: biological processes
DEG: differentially expressed gene
EMT: epithelial–mesenchymal transition
GEO: Gene Expression Omnibus
MF: molecular functions
PCRC: primary colorectal cancer
PPI: protein–protein interaction
RNAi: RNA interference
