# Supplementary material for: CLCA4 and MS4A12 as the significant gene biomarkers of primary colorectal cancer
Source: Biosci Rep. 2020 Aug 20;40(8):BSR20200963. doi: 10.1042/BSR20200963 (PMC7441370; doi:10.1042/BSR20200963)
Supplement: Supplementary Figures S1-S5 [file BSR-2020-0963_supp.pdf]

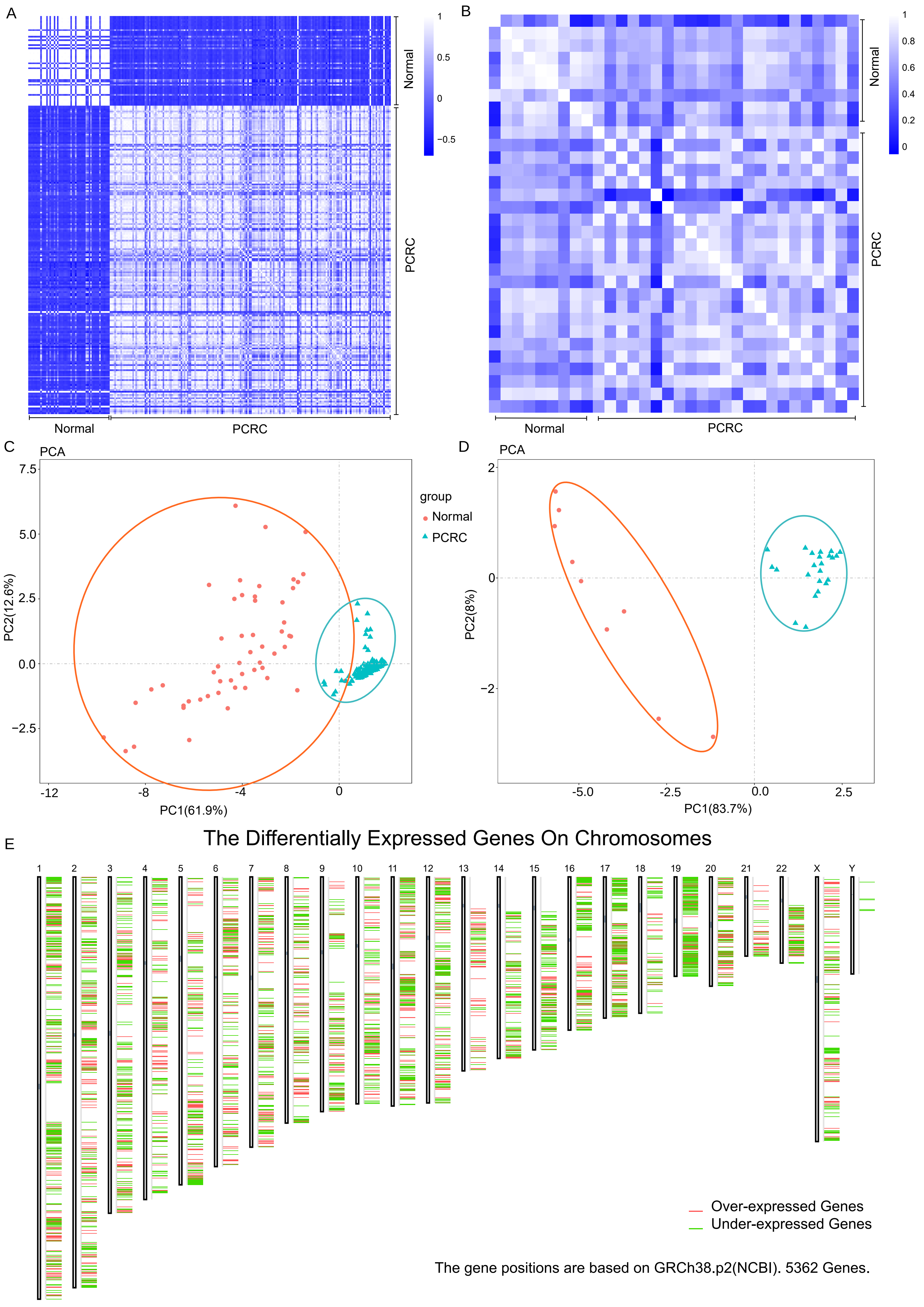
A
Normal
PCRC
1
0.5
0
−0.5
B
1
0.8
0.6
0.4
0.2
0
C
PCA
group
Normal
PCRC
PC2(12.6%)
PC1(61.9%)
D
PCA
PC2(8%)
PC1(83.7%)
E
The Differentially Expressed Genes On Chromosomes
1 2 3 4 5 6 7 8 9 10 11 12 13 14 15 16 17 18 19 20 21 22 X Y
Over-expressed Genes
Under-expressed Genes
The gene positions are based on GRCh38.p2(NCBI). 5362 Genes.

Figure S1: Verification for repeatability of intra-group data and the differentially expressed genes on chromosomes.

(A) Heat map indicating the strong correlations among samples in the PCRC group, and also strong correlations among samples in the control group in the GSE41258 via the Pearson’s correlation test. The 0<correlation<1 showed that there is a positive correlation. The -1<correlation<0 showed that there is a negative correlation. The larger the absolute value is, the stronger the correlation is. (B) Heat map indicating the strong correlations among samples in the PCRC group, and also strong correlations among samples in the control group in the GSE81558 via the Pearson’s correlation test. The 0<correlation<1 showed that there is a positive correlation. The -1<correlation<0 showed that there is a negative correlation. The larger the absolute value is, the stronger the correlation is. (C) The PCA manifested that the repeatability of the data in GSE41258 was fine. The distances between per samples in the PCRC group were close, and the distances between per samples in the control group were also close in the dimension of PC1. (D) The PCA manifested that the repeatability of the data in GSE81558 was fine. The distances between per samples in the PCRC group were close, and the distances between per samples in the control group were also close in the dimension of PC1. (E) The differentially expressed genes on chromosomes between PCRC and normal colorectal tissue.

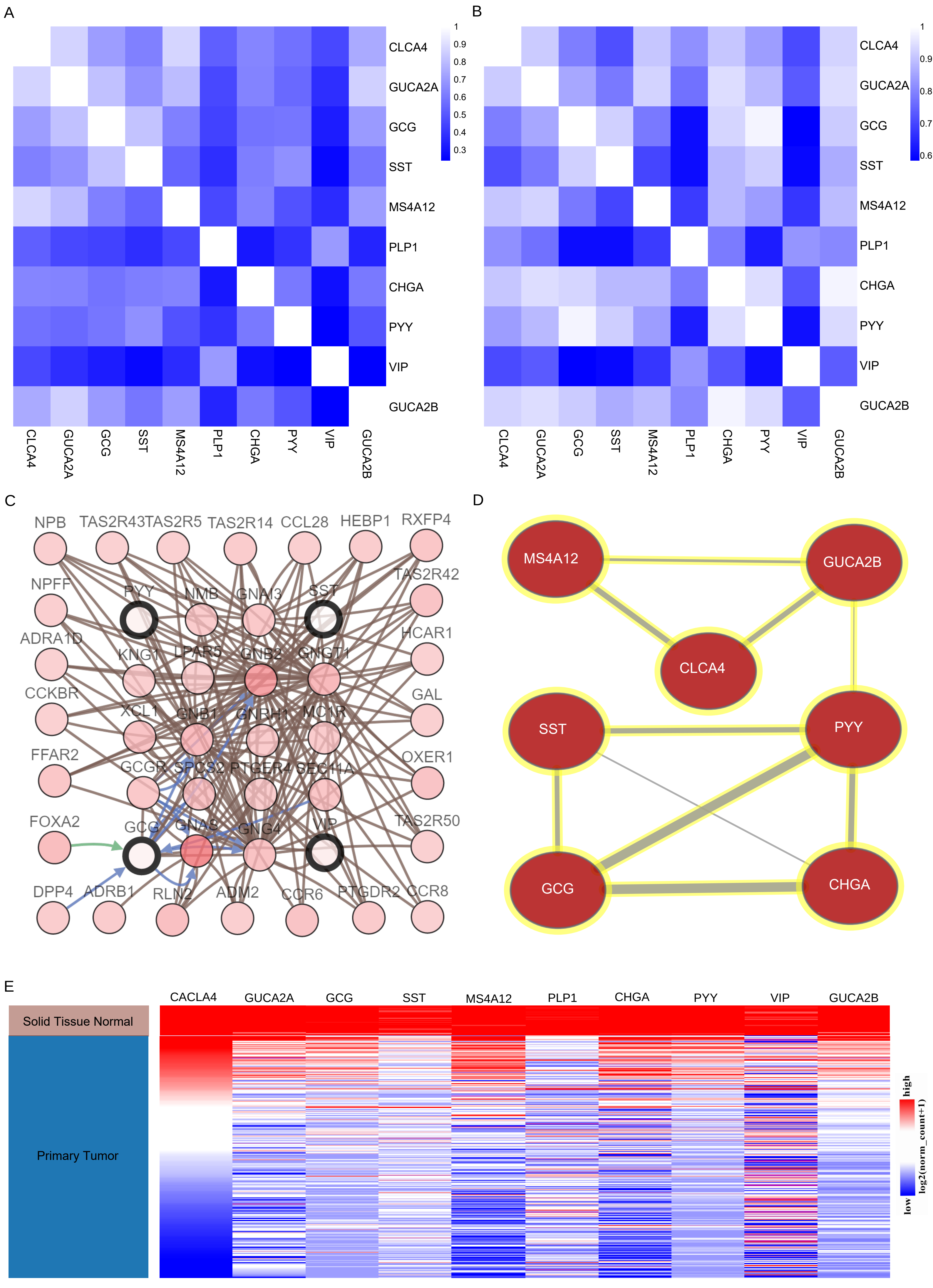
A
B
CLCA4
GUCA2A
GCG
SST
MS4A12
PLP1
CHGA
PYY
VIP
GUCA2B
1
0.9
0.8
0.7
0.6
0.5
0.4
0.3
C
NPB
TAS2R43
TAS2R5
TAS2R14
CCL28
HEBP1
RXFP4
NPFF
PYY
NMB
GNAI3
SST
TAS2R42
ADRA1D
KNG1
LPAR5
GNB2
GNGT1
HCAR1
CCKBR
XCL1
GNB1
GNRH1
MC1R
GAL
FFAR2
GCGR
SPCS2
PTGER4
SEC11A
OXER1
FOXA2
GCG
GNAS
GNG4
VIP
TAS2R50
DPP4
ADRB1
RLN2
ADM2
CCR6
PTGDR2
CCR8
D
MS4A12
GUCA2B
CLCA4
SST
PYY
GCG
CHGA
E
Solid Tissue Normal
Primary Tumor
CACLA4
GUCA2A
GCG
SST
MS4A12
PLP1
CHGA
PYY
VIP
GUCA2B
high
low
log2(norm_count+1)

Figure S2: The correlation analysis among the hub genes and the UCSC analysis. (A) Heat maps showing the correlations between hub genes in the GSE41258datasets. (B) Heat maps showing the correlations between hub genes in the GSE81558datasets. The color reflects the intensity of the correlation. The 0<correlation<1 showed that there is a positive correlation. The -1<correlation<0 showed that there is a negative correlation. The larger the absolute value is, the stronger the correlation is. (C) PYY, SST, GCG, and VIP existed simultaneously in the co-expression network via cBioPortal. (D) Through the analysis of Coexpedia, there were strong interactions among PYY, SST, GCG, CHGA, CLCA4, GUCA2B, and MS4A12. (E) Heat map showed that the expressions of all the hub genes were lower in the PCRC samples than the control samples.

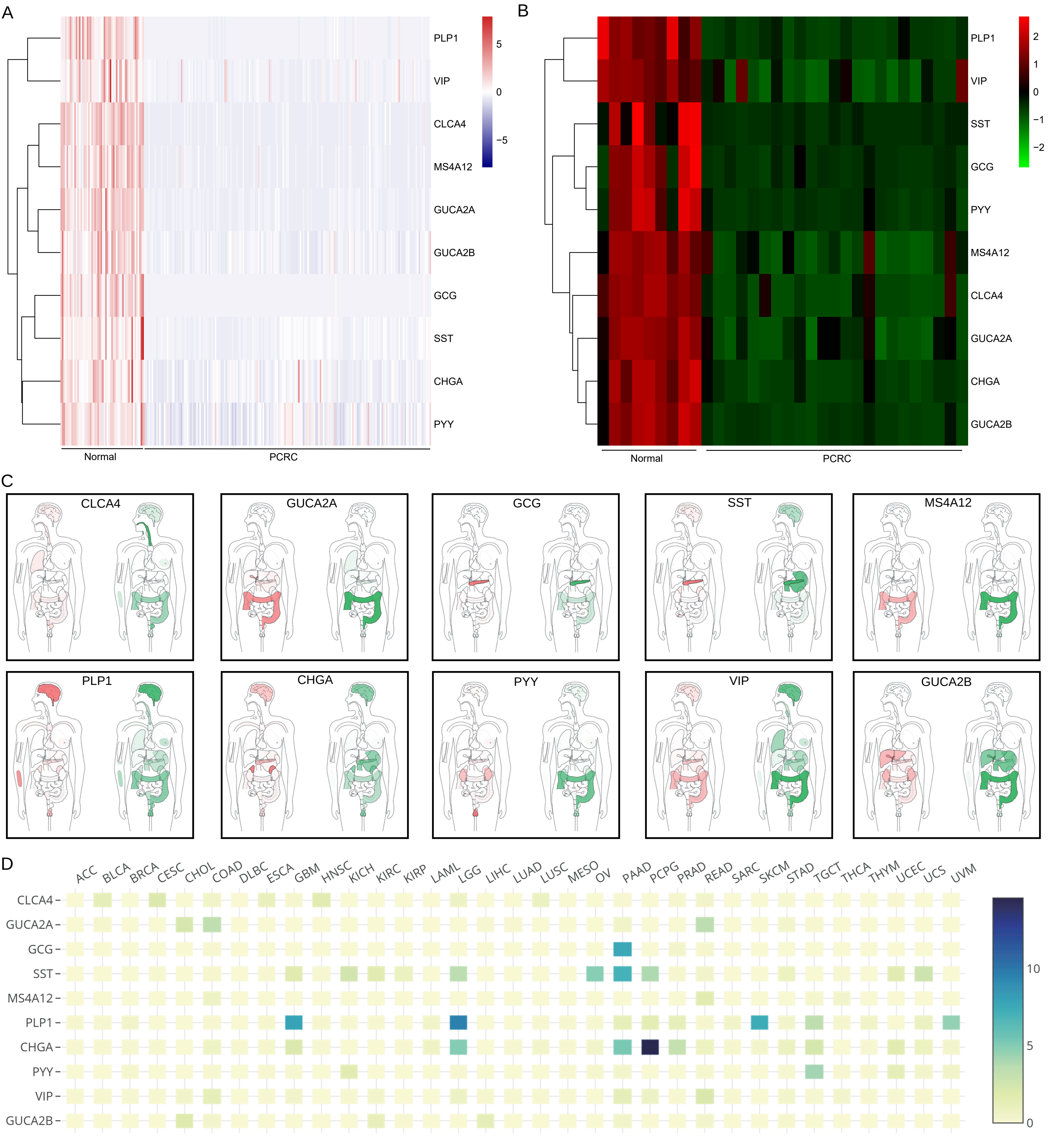

A
PLP1
VIP
CLCA4
MS4A12
GUCA2A
GUCA2B
GCG
SST
CHGA
PYY
5
0
−5
Normal
PCRC
B
PLP1
VIP
SST
GCG
PYY
MS4A12
CLCA4
GUCA2A
CHGA
GUCA2B
2
1
0
−1
−2
Normal
PCRC
C
CLCA4
GUCA2A
GCG
SST
MS4A12
PLP1
CHGA
PYY
VIP
GUCA2B
D
ACC BLCA BRCA CESC CHOL COAD DLBC ESCA GBM HNSC KICH KIRC KIRP LAML LGG LIHC LUAD LUSC MESO OV PAAD PCPG PRAD READ SARC SKCM STAD TGCT THCA THYM UCEC UCS UVM
CLCA4
GUCA2A
GCG
SST
MS4A12
PLP1
CHGA
PYY
VIP
GUCA2B
10
5
0

Figure S3: The expression analysis of the hub genes.

(A) A hierarchical clustering heat-map of the hub genes from PCRC and normal colorectal tissue from the GSE41258 dataset. The X-axis represents sample symbols (from left to right: samples of normal colorectal tissue and samples of PCRC tissue); the Y-axis represents hub genes. Blue, low expression; white, medium expression; and pink, high expression. (B) A hierarchical clustering heat-map of the hub genes from PCRC and normal colorectal tissue from the GSE81558 dataset. The X-axis represents sample symbols (from left to right: samples of normal colorectal tissue and samples of PCRC tissue); the Y-axis represents hub genes. Green, low expression; black, medium expression; and red, high expression. (C) The expression of hub genes (CLCA4, GUCA2A, GCG, SST, MS4A12, PLP1, CHGA, PYY, VIP, and GUCA2B) in the colorectum was higher in the normal individuals compared with the tumor samples. (D) The comparison of expressions of all hub genes in the various tumors. ACC: Adrenocortical carcinoma; BLCA: Bladder Urothelial Carcinoma; BRCA: Breast invasive carcinoma; CESC: Cervical squamous cell carcinoma and endocervical adenocarcinoma; CHOL: Cholangio carcinoma; COAD: Colon adenocarcinoma; DLBC: Lymphoid Neoplasm Diffuse Large B-cell Lymphoma; ESCA: Esophageal carcinoma; GBM: Glioblastoma multiforme; HNSC: Head and Neck squamous cell carcinoma; KICH: Kidney Chromophobe; KIRC: Kidney renal clear cell carcinoma; KIRP: Kidney renal papillary cell carcinoma; LAML: Acute Myeloid Leukemia; LGG: Brain Lower Grade Glioma; LIHC: Liver hepatocellular carcinoma; LUAD: Lung adenocarcinoma; LUSC: Lung squamous cell carcinoma; OA: Ovarian serous cystadenocarcinoma; PAAD: Pancreatic adenocarcinoma; PCPG: Pheochromocytoma and Paraganglioma; PRAD: Prostate adenocarcinoma; READ: Rectum adenocarcinoma; SARC: Sarcoma; SKCM: Skin Cutaneous Melanoma;

STAD: Stomach adenocarcinoma; TGCT: Testicular Germ Cell Tumors; THCA: Thyroid carcinoma; THYM: Thymoma; UCEC: Uterine Corpus Endometrial Carcinoma; UCS: Uterine Carcinosarcoma.

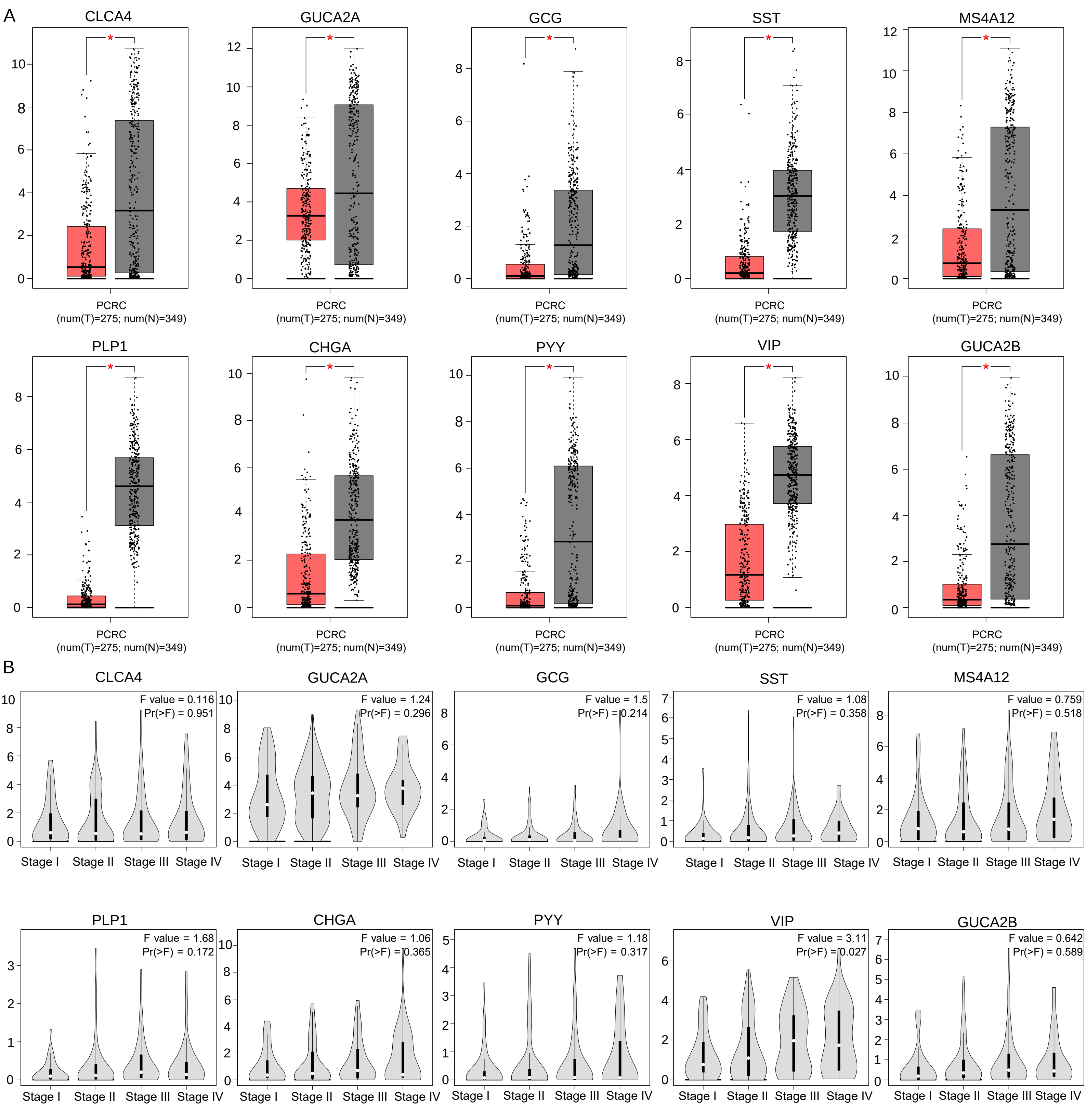
A
CLCA4
GUCA2A
GCG
SST
MS4A12
PLP1
CHGA
PYY
VIP
GUCA2B
*
PCRC
(num(T)=275; num(N)=349)
B
CLCA4
F value = 0.116
Pr(>F) = 0.951
GUCA2A
F value = 1.24
Pr(>F) = 0.296
GCG
F value = 1.5
Pr(>F) = 0.214
SST
F value = 1.08
Pr(>F) = 0.358
MS4A12
F value = 0.759
Pr(>F) = 0.518
PLP1
F value = 1.68
Pr(>F) = 0.172
CHGA
F value = 1.06
Pr(>F) = 0.365
PYY
F value = 1.18
Pr(>F) = 0.317
VIP
F value = 3.11
Pr(>F) = 0.027
GUCA2B
F value = 0.642
Pr(>F) = 0.589
Stage I
Stage II
Stage III
Stage IV

Figure S4: The box plots of expression level of hub genes and the violin plots of pathological stage.

(A) The comparison of expressions of all hub genes between PCRC and normal colorectal samples. (B) The relationship between expression of hub genes (CLCA4, GUCA2A, GCG, SST, MS4A12, PLP1, CHGA, PYY, VIP, and GUCA2B) and pathological stage.

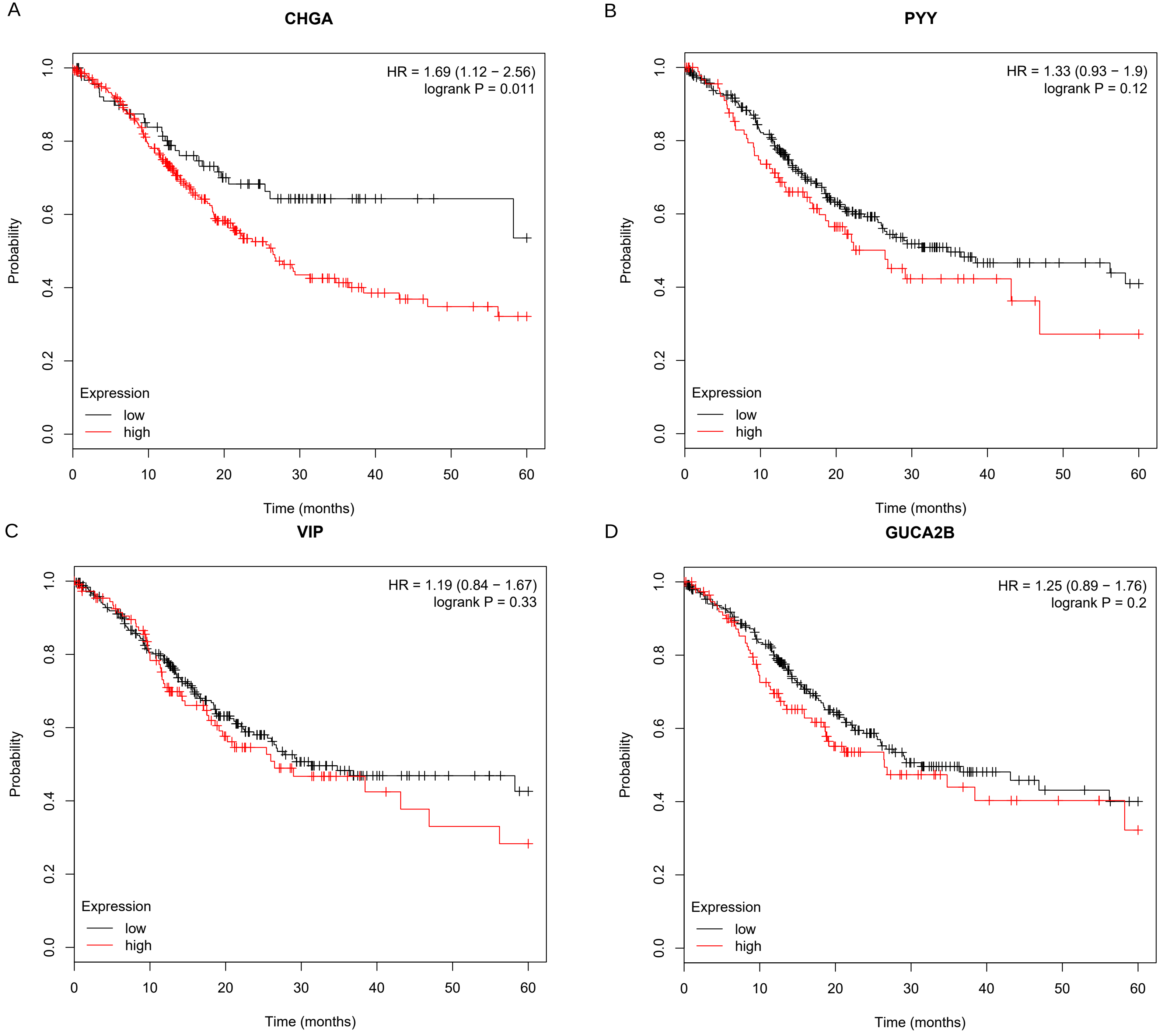
A
CHGA
HR = 1.69 (1.12 − 2.56)
logrank P = 0.011
Probability
Time (months)
Expression
low
high
B
PYY
HR = 1.33 (0.93 − 1.9)
logrank P = 0.12
Probability
Time (months)
Expression
low
high
C
VIP
HR = 1.19 (0.84 − 1.67)
logrank P = 0.33
Probability
Time (months)
Expression
low
high
D
GUCA2B
HR = 1.25 (0.89 − 1.76)
logrank P = 0.2
Probability
Time (months)
Expression
low
high

Figure S5: The overall survival Kaplan-Meier of another four hub genes.

(A) CHGA, (B) PYY, (C) VIP, (D) GUCA2B
